# Antiseptic Treatment of Intestinal Affections

**Published:** 1888-02

**Authors:** 


ANTISEPTIC TREATMENT OF INTESTINAL AFFECTIONS —In an article on "Intestinal Antiseptics," by D. N. Kinsman, M. D., appearing in the *Journal of the American Medical Association*, July 3d, 1886, the author points out that the natural process of fermentation and putrefaction going on in normal digestion are so changed in dyspepsia and other forms of intestinal diseases as to produce poisonous alkaloids which are the cause of the symptoms developed in such disorders.

The researches of Prof. Vaughn, of the University of Michigan, in which tyro-toxicon has been shown to be the cause of ice cream poisoning, which are still fresh in the minds of medical readers, have thrown still more light on the etiology of intestinal antisepsis as a method of treatment.

To facilitate such treatment we learn that Parke, Davis & Co. have recently added to their list an intestinal antiseptic pill, the formula of which is as follows: Mercury, protiodide, 1-8 gr.; podophyllin, 1 16 gr.; aloin, 1-16 gr.; ext. nux vomica, 1-16 gr.; ext. henbane, 1.16 gr.

